# Effect of Perioperative Ketamine and Esketamine on Postoperative Fatigue: A Systematic Review and Meta-Analysis of Randomized Controlled Trials

**DOI:** 10.3390/medicina62061156

**Published:** 2026-06-14

**Authors:** Mohammed Al Subhi, Mohammed Al Yousufi, Hassan Alnajdi, Athir Al-Balushi, Ibrahim K. Al Alawi, Saleh Al Abri, Younis Al-Mufargi

**Affiliations:** 1 School of Medicine, Medical Sciences and Nutrition, University of Aberdeen, Foresterhill, Aberdeen AB25 2ZD, UK; m.alsubhi.23@abdn.ac.uk (M.A.S.); m.alyousufi.23@abdn.ac.uk (M.A.Y.); h.alnajdi.23@abdn.ac.uk (H.A.); 2 Department of Anesthesia, Sohar Hospital, Ministry of Health, Sohar 321, Oman; athiryousuf36@gmail.com; 3 Department of General Surgery, Medical City for Military and Security Services, Highway Muscat Street, Muscat 121, Oman; alalawiibrahim@yahoo.com; 4 Department of Anesthesia, Medical City for Military and Security Services, Highway Muscat Street, Muscat 121, Oman

**Keywords:** postoperative fatigue, ketamine, esketamine, perioperative care, meta-analysis

## Abstract

*Background and Objectives*: Postoperative fatigue (POF) is a frequent complication that negatively affects recovery and quality of life following surgery. Ketamine and esketamine, as N-methyl-D-aspartate (NMDA) receptor antagonists, may reduce postoperative fatigue; however, current evidence remains inconclusive. This systematic review and meta-analysis aimed to evaluate the effects of perioperative ketamine and esketamine on postoperative fatigue in adult surgical patients. *Materials and Methods*: A systematic search of major electronic databases was conducted to identify randomized controlled trials comparing perioperative ketamine or esketamine with placebo or standard care in adult surgical patients. Fatigue outcomes during the first postoperative week were analyzed using random-effects meta-analyses and reported as standardized mean differences (SMDs) with 95% confidence intervals (CIs). Subgroup analyses were performed according to NMDA antagonist type, surgical procedure, and administration regimen. Sensitivity analyses included exclusion of studies using patient-controlled intravenous analgesia (PCIA) and leave-one-out analyses. Risk of bias was assessed using the Cochrane Risk of Bias 2 tool, and certainty of evidence was evaluated using the GRADE approach. *Results*: Eight randomized controlled trials (n = 906 participants) were included in the qualitative and quantitative synthesis. No significant overall reduction in postoperative fatigue was observed at POD1 (SMD −0.37, 95% CI −0.87 to 0.13), although esketamine demonstrated a significant benefit in subgroup analysis. Ketamine/esketamine significantly reduced postoperative fatigue at POD3 (SMD −0.58, 95% CI −0.86 to −0.30) and POD7 (SMD −0.38, 95% CI −0.65 to −0.12). Subgroup analyses demonstrated greater reductions in fatigue among patients undergoing colorectal surgery. Sensitivity analyses excluding PCIA studies and leave-one-out analyses confirmed the robustness of the findings at POD3 and POD7. The certainty of evidence was rated as low for POD1 and moderate for POD3 and POD7. *Conclusions*: Perioperative ketamine and esketamine were associated with reduced postoperative fatigue from POD3 onward in adult surgical patients, with more consistent benefits observed in colorectal surgery populations. Further high-quality studies are needed to determine the clinical significance of these findings and their impact on postoperative recovery outcomes.

## 1. Introduction

Postoperative fatigue (POF) is a common complaint of patients after surgery. It is characterized by low energy levels and delayed return to normal activities. Significant POF is a common complaint after surgery with the prevalence being as high as 37% in the first postoperative day [1]. Fatigue after surgery is considered a normal physiological phenomenon. However, there is limited evidence on the root cause of this problem. Some theories suggest that because surgery puts significant stress on the body, this leads to hormonal changes that contribute to the feeling of tiredness [2]. POF is associated with numerous postoperative complications that may hinder convalescence. Jensen et al. (2011) [3] found that there is a correlation between POF, reduced physical activity, and delayed mobilization [3]; this in turn is linked to adverse postoperative complications and higher mortality [4]. Fatigue is quantified in clinical trials using different scales (most notably the ICFS fatigue score) [2,5]. Fatigue is a modifiable postoperative complication as demonstrated by numerous trials [5,6]. Therefore, it is important to address this problem to improve recovery and outcomes after surgery.

Ketamine and, its more potent S-enantiomer, esketamine have been widely implemented in perioperative care for their analgesic and anesthetic properties [7]. They work as N-methyl-D-aspartate receptor antagonists (NMDA), reducing the excitatory effects of the CNS neurotransmitter glutamate leading to reduced central sensitization to pain [8]. Evidence suggests that ketamine and esketamine are very effective for perioperative anesthesia and analgesia. Moreover, they can improve postoperative sleep quality, reduce the use of opiates, and improve cognitive function, all of which contribute to improving overall postoperative recovery [9,10,11]. Additionally, they have demonstrated high efficacy in the management of psychiatric conditions including depression and anxiety [12]. Due to its inhibitory effects on the CNS, retrospective studies have shown that ketamine can demonstrate some efficacy in the treatment of resistant status epilepticus, although the quality of evidence on this matter is poor and requires more investigation [13].

Several randomized clinical trials suggested that ketamine and esketamine can contribute to improving POF. In this systematic review and meta-analysis, the objective was to collect, review, and synthesize evidence on the effect of ketamine and esketamine on postoperative fatigue.

## 2. Materials and Methods

**Study Design:** This study was conducted as a systematic review and meta-analysis in accordance with the PRISMA 2020 guidelines and was prospectively registered in PROSPERO (registration number: CRD420251166574). The objective was to evaluate the efficacy of ketamine and esketamine in reducing POF, measured using validated patient-reported outcome instruments over the first postoperative week.

**Eligibility Criteria:** Inclusion Criteria: Studies were included if they met the following criteria: Population: Adult patients (≥18 years) undergoing any surgical procedure. Intervention: Perioperative administration of ketamine or esketamine. Comparison: Placebo or standard care. Outcomes: Assessment of POF within the first week after surgery. Study Design: Randomized controlled trials (RCTs).

Exclusion Criteria: Pediatric populations, studies not reporting fatigue outcomes, non-comparative studies (e.g., case series, editorials), animal studies, and conference abstracts without full data.

**Search Strategy:** A comprehensive literature search was conducted in the databases PubMed, OVID (MEDLINE, EMBASE, Central Cochrane Library), Web of Science, and Scopus, from inception to 12 September 2025. Search terms included combinations of: (“ketamine” OR “esketamine”) AND (“postoperative fatigue” OR “fatigue” OR “fatigue syndrome” OR “post-surgical fatigue”) AND (“fatigue scale” OR “fatigue assessment” OR “Identity–Consequence Fatigue Scale” OR “ICFS” OR “Christensen”) AND (“surgery” OR “postoperative” OR “perioperative”). Reference lists of included studies and relevant reviews were also screened manually for additional eligible studies. The full search strategy is available in the Supplementary Materials (Table S1).

**Study Selection:** Two independent reviewers (M.A.Y. and H.A.) screened titles and abstracts. Full texts of potentially eligible studies were retrieved and assessed for inclusion. Disagreements were resolved through discussion with a third reviewer.

**Quality Assessment:** Two reviewers (M.A.S. and Y.A.) assessed the methodological quality of included studies using the Cochrane Risk of Bias Tool (RoB 2.0). Domains assessed included randomization, allocation concealment, blinding, incomplete outcome data, selective reporting, and other biases. Studies were classified as low risk of bias, some concerns, or high risk of bias according to RoB 2.0 guidance.

**Data Extraction:** Data extraction was conducted using a standardized form to systematically collect relevant information from each study. This included study characteristics (title, authors, year, country, and design), patient population details (type of surgery, sample size, age, and sex), details of the intervention (dose, route, and timing of ketamine or esketamine), comparator information. Postoperative fatigue outcomes were extracted as mean and standard deviation values at postoperative days (PODs) 1, 3, and 7 across different fatigue assessment scales. Additionally, statistical data such as confidence intervals, *p*-values, and effect sizes were extracted. Where reported, objective recovery outcomes (e.g., hospital length of stay and post-anesthesia care unit (PACU)/early recovery unit duration) were also extracted. Studies not published in English were translated using automated translation software (Google Translate). Two reviewers independently (M.A.S. and A.A.) performed the data extraction to ensure accuracy and consistency.

**Data Synthesis and Statistical Analysis:** All meta-analyses were conducted using R (meta package). Continuous outcomes were pooled using standardized mean differences (SMDs) with 95% confidence intervals (CIs), as different fatigue assessment scales were used across the included studies. When multiple fatigue assessment scales were reported within a study, data from the Identity–Consequence Fatigue Scale (ICFS) were preferentially extracted for analysis. A random-effects model (DerSimonian and Laird method) was applied because of anticipated clinical and methodological heterogeneity among studies. Quantitative synthesis was performed for studies reporting postoperative fatigue outcomes at postoperative days (PODs) 1, 3, and 7. Subgroup analyses were conducted according to the type of NMDA receptor antagonist (ketamine vs esketamine), surgical procedure, and administration regimen.

Statistical heterogeneity was assessed using the *I*^2^ statistic and interpreted according to Cochrane guidance as follows: 0–40% (might not be important), 30–60% (may represent moderate heterogeneity), 50–90% (may represent substantial heterogeneity), and 75–100% (considerable heterogeneity). Leave-one-out (LOO) sensitivity analyses were performed to evaluate whether the pooled effect estimates were disproportionately influenced by any individual study, including studies assessed as having a high risk of bias. Additional sensitivity analyses were conducted by excluding studies using patient-controlled intravenous analgesia (PCIA). To account for multiplicity across postoperative time point comparisons (POD1, POD3, and POD7), Holm–Bonferroni adjustment was applied. Objective recovery outcomes were summarized qualitatively when reported, as variability in reporting methods and the limited number of contributing studies precluded meta-analysis.

## 3. Results

**Figure 1:**
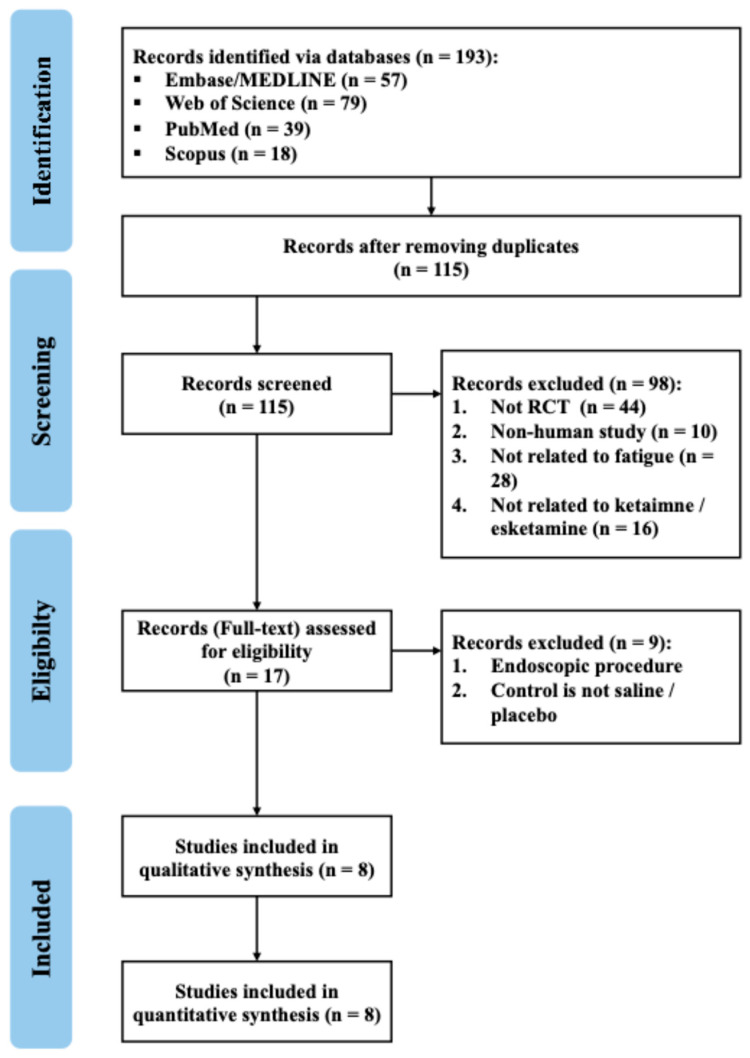
PRISMA flow diagram of study selection.

A comprehensive literature search through the databases yielded 193 records, of which 115 remained after removing duplicates. After title and abstract screening, 17 full-text articles were assessed for eligibility. Ultimately, eight randomized controlled trials (RCTs) were included in the qualitative and quantitative synthesis [5,6,14,15,16,17,18,19]. The study selection process is detailed in the PRISMA flow diagram (Figure 1).

The included studies were conducted between 2012 and 2024, with most originating from China. Across the eight randomized controlled trials, 906 participants were enrolled (456 intervention and 450 control). Mean participant age ranged from 45.6 to 72.3 years, and sex distribution varied according to the surgical population. Surgical procedures included colorectal, gastric, breast, thoracic, orthopedic, and gynecological surgery. Interventions involved perioperative ketamine or esketamine administered via intravenous bolus, infusion, or patient-controlled intravenous analgesia (PCIA), compared with placebo or standard care (Table 1).

**Figure 2:**
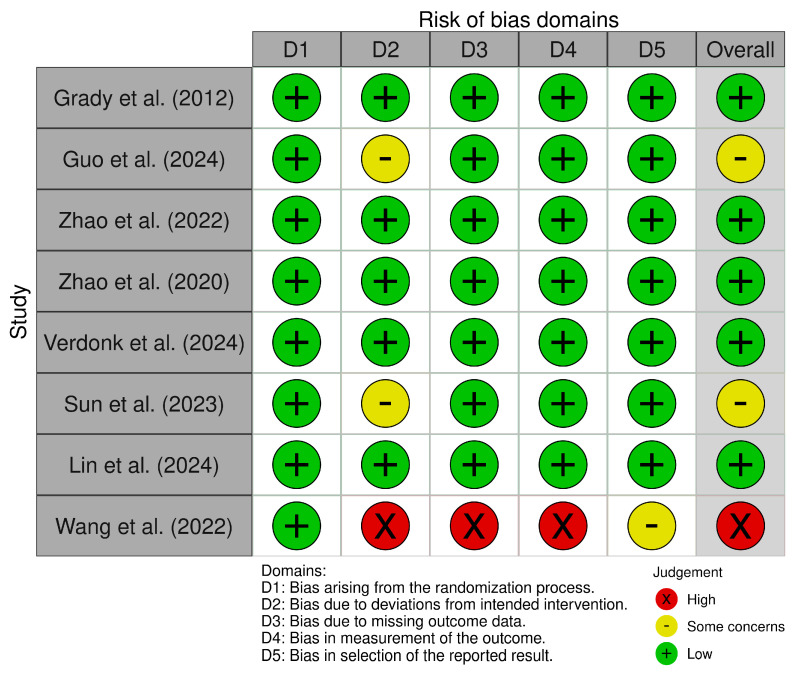
Risk of bias assessment for included RCTs [5,6,14,15,16,17,18,19].

Risk of bias assessment using the Cochrane RoB 2.0 tool revealed that five of the eight included RCTs had low risk of bias. One study (Wang et al., 2023) [11] was judged to have high risk of bias due to issues in outcome measurement and missing data, while two studies showed “some concerns” due to deviations from intended intervention or selective reporting (Figure 2).

**Figure 3:**
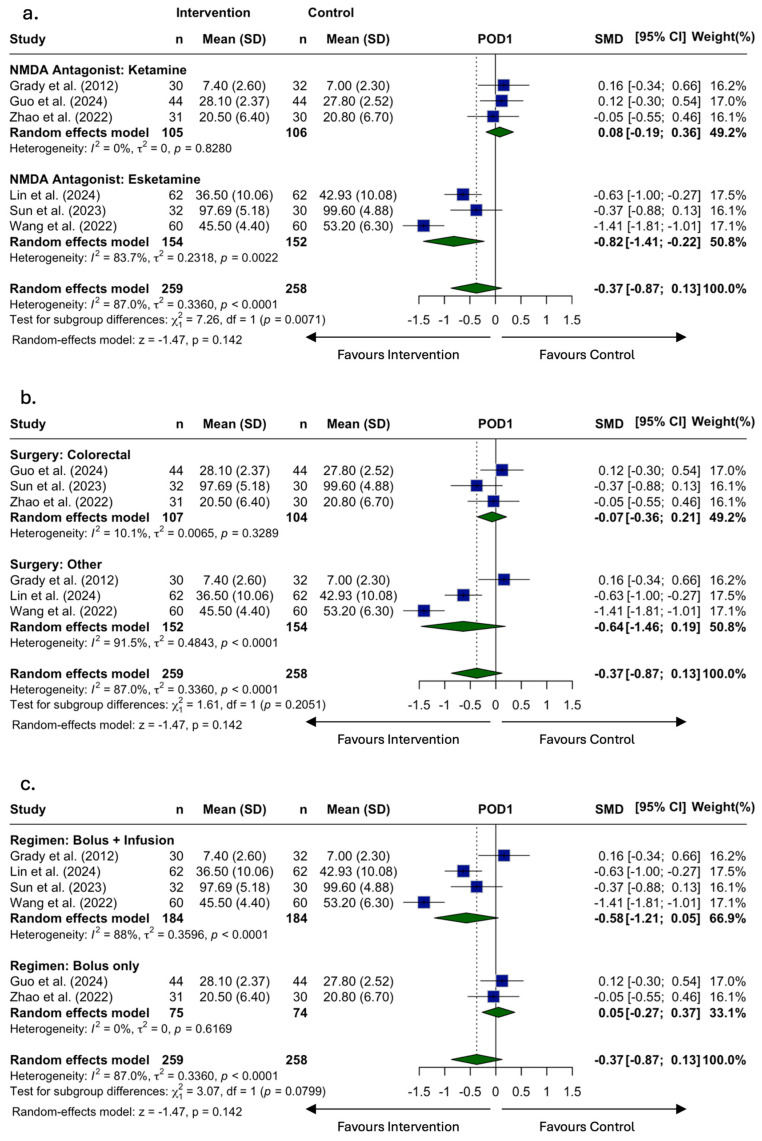
Forest plots demonstrating the effects of ketamine/esketamine on postoperative fatigue at postoperative day 1 (POD1). Subgroup analyses were performed according to (**a**) NMDA antagonist type [5,6,14,15,18,19], (**b**) surgical procedure [5,6,14,15,18,19], and (**c**) administration regimen [5,6,14,15,18,19].

At POD1, data from 517 participants (259 intervention and 258 control) were available (Figure 3). The pooled analysis demonstrated no significant reduction in postoperative fatigue with ketamine/esketamine compared with control (SMD −0.37, 95% CI −0.87 to 0.13; *p* = 0.142), with substantial heterogeneity across studies (*I*^2^ = 87.0%, *p* < 0.0001). Subgroup analysis according to NMDA antagonist type (Figure 3a) showed that esketamine significantly reduced fatigue scores (SMD −0.82, 95% CI −1.41 to −0.22), whereas ketamine showed no significant effect (SMD 0.08, 95% CI −0.19 to 0.36); the between-subgroup difference was statistically significant (*p* = 0.0071). Stratification by surgery type (Figure 3b) demonstrated no significant benefit in either colorectal surgery (SMD −0.07, 95% CI −0.36 to 0.21) or other surgical procedures (SMD −0.64, 95% CI −1.46 to 0.19), with no significant subgroup interaction (*p* = 0.2051). Similarly, subgroup analysis according to administration regimen (Figure 3c) showed no significant subgroup difference between bolus plus infusion (SMD −0.58, 95% CI −1.21 to 0.05) and bolus-only administration (SMD 0.05, 95% CI −0.27 to 0.37; subgroup *p* = 0.0799).

**Figure 4:**
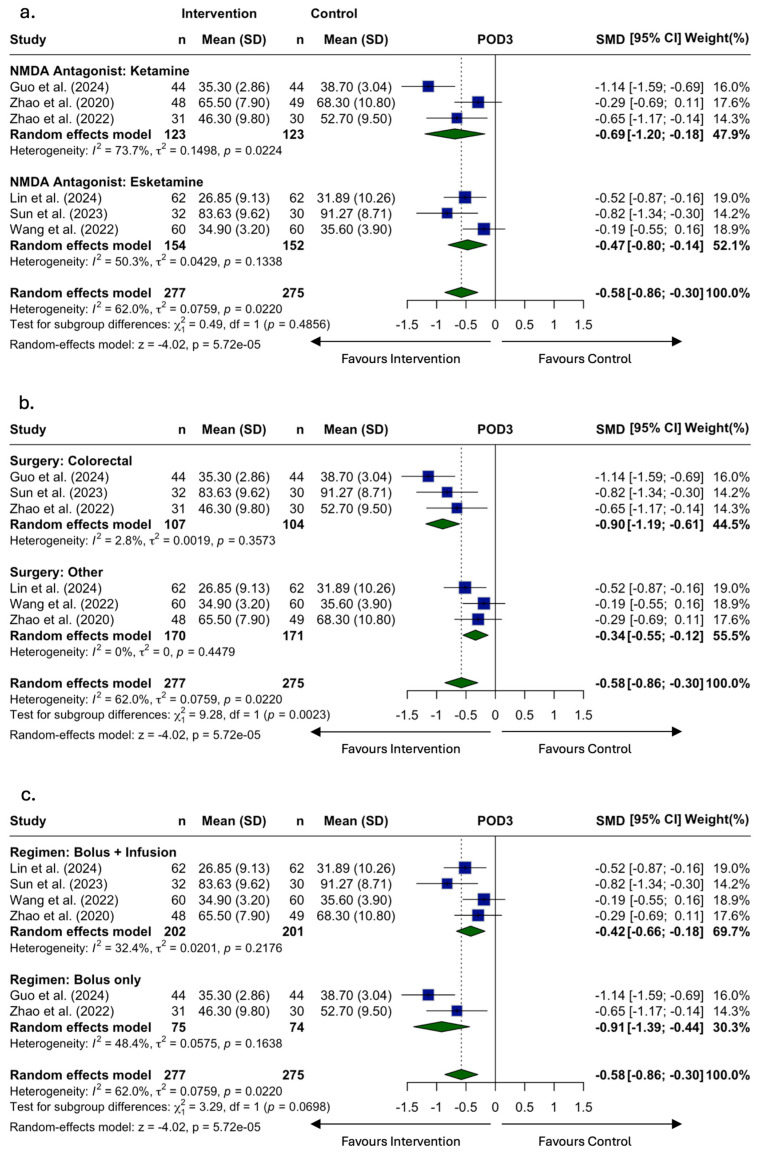
Forest plots demonstrating the effects of ketamine/esketamine on postoperative fatigue at postoperative day 3 (POD3). Subgroup analyses were performed according to (**a**) NMDA antagonist type [5,6,15,16,18,19], (**b**) surgical procedure [5,6,15,16,18,19], and (**c**) administration regimen [5,6,15,16,18,19].

At POD3, six studies comprising 552 participants (277 intervention and 275 control) were included (Figure 4). The pooled analysis demonstrated a significant reduction in postoperative fatigue favoring ketamine/esketamine over control (SMD −0.58, 95% CI −0.86 to −0.30; *p* < 0.001), with moderate heterogeneity (*I*^2^ = 62.0%, *p* = 0.0220). Subgroup analysis by NMDA antagonist type (Figure 4a) showed significant reductions in fatigue with both ketamine (SMD −0.69, 95% CI −1.20 to −0.18) and esketamine (SMD −0.47, 95% CI −0.80 to −0.14), without a significant subgroup difference (*p* = 0.4856). When analyzed according to surgery type (Figure 4b), the reduction in fatigue was greater in patients undergoing colorectal surgery (SMD −0.90, 95% CI −1.19 to −0.61) than in those undergoing other surgical procedures (SMD −0.34, 95% CI −0.55 to −0.12), with a significant subgroup interaction (*p* = 0.0023). Both bolus plus infusion (SMD −0.42, 95% CI −0.66 to −0.18) and bolus-only regimens (SMD −0.91, 95% CI −1.39 to −0.44) were associated with significant reductions in fatigue (Figure 4c), although the difference between regimens did not reach statistical significance (*p* = 0.0698).

**Figure 5:**
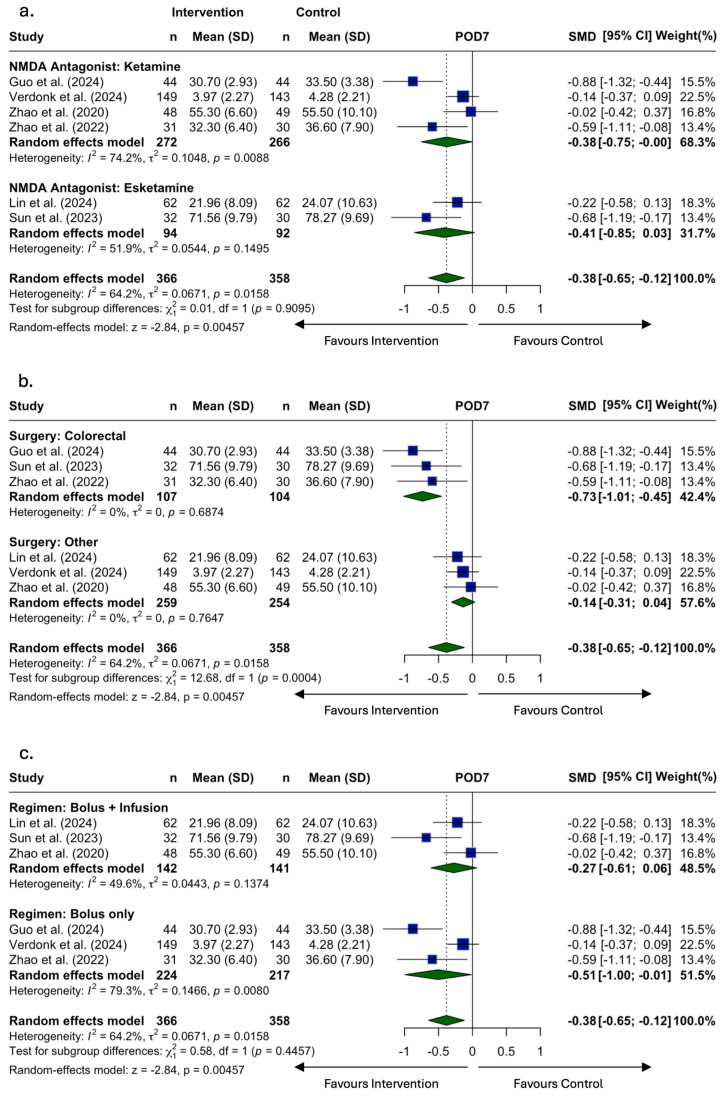
Forest plots demonstrating the effects of ketamine/esketamine on postoperative fatigue at postoperative day 7 (POD7). Subgroup analyses were performed according to (**a**) NMDA antagonist type [5,6,15,17,16,18], (**b**) surgical procedure [5,6,15,17,16,18], and (**c**) administration regimen [5,6,15,17,16,18].

At POD7, six studies involving 724 participants (366 intervention and 358 control) were analyzed (Figure 5). Ketamine/esketamine administration remained associated with a significant reduction in postoperative fatigue compared with control (SMD −0.38, 95% CI −0.65 to −0.12; *p* = 0.0046), with moderate heterogeneity (*I*^2^ = 64.2%, *p* = 0.0158). Subgroup analysis according to NMDA antagonist type (Figure 5a) demonstrated comparable effects for ketamine (SMD −0.38, 95% CI −0.75 to −0.00) and esketamine (SMD −0.41, 95% CI −0.85 to 0.03), with no significant subgroup difference (*p* = 0.9095). Stratification by surgery type (Figure 5b) showed a significant reduction in fatigue among patients undergoing colorectal surgery (SMD −0.73, 95% CI −1.01 to −0.45), whereas no significant effect was observed in other surgical procedures (SMD −0.14, 95% CI −0.31 to 0.04); the subgroup interaction was significant (*p* = 0.0004). In the regimen-based subgroup analysis (Figure 5c), bolus-only administration significantly reduced postoperative fatigue (SMD −0.51, 95% CI −1.00 to −0.01), whereas bolus plus infusion did not reach statistical significance (SMD −0.27, 95% CI −0.61 to 0.06). However, no significant subgroup difference between administration regimens was identified (*p* = 0.4457).

**Figure 6:**
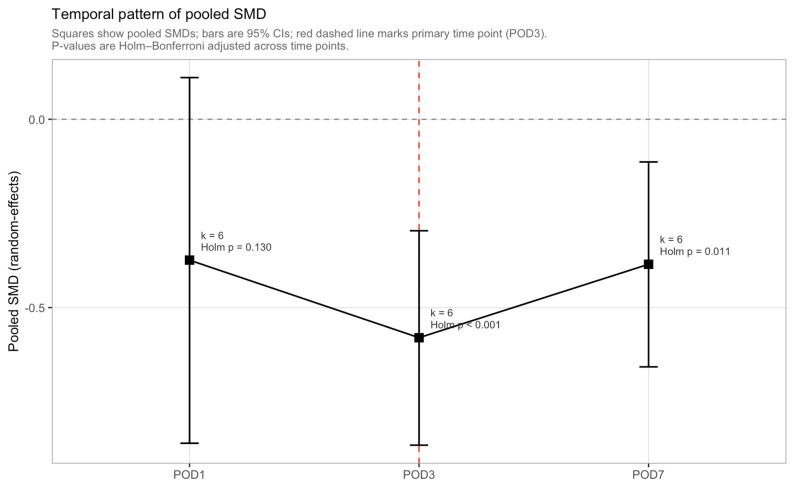
Temporal pattern of pooled standardized mean differences (SMDs) for postoperative fatigue across postoperative days (PODs) 1, 3, and 7. Squares represent pooled SMD estimates from random-effects models, and error bars indicate 95% confidence intervals (CIs). Holm–Bonferroni-adjusted *p*-values are shown for each time point. The red dashed line denotes the primary postoperative time point (POD3).

To account for multiple comparisons across postoperative time points, Holm–Bonferroni adjustment was applied to the pooled analyses at POD1, POD3, and POD7 (Figure 6). Following adjustment, the reduction in postoperative fatigue remained statistically significant at POD3 (Holm-adjusted *p* < 0.001) and POD7 (Holm-adjusted *p* = 0.011), whereas the effect at POD1 was not significant (Holm-adjusted *p* = 0.130). The temporal pattern demonstrated the greatest reduction in postoperative fatigue at POD3, followed by a sustained but attenuated effect at POD7.

Sensitivity analyses excluding studies using patient-controlled intravenous analgesia (PCIA) were performed to evaluate the robustness of the primary findings (Figures S1–S3). After exclusion of these studies, no significant reduction in postoperative fatigue was observed at POD1 (SMD −0.02, 95% CI −0.26 to 0.22; *I*^2^ = 0%). In contrast, ketamine/esketamine remained associated with significantly lower postoperative fatigue scores at POD3 (SMD −0.72, 95% CI −1.10 to −0.34; *I*^2^ = 61.9%) and POD7 (SMD −0.43, 95% CI −0.76 to −0.10; *I*^2^ = 70.9%). Overall, the sensitivity analyses demonstrated findings consistent with the primary analyses, supporting the robustness of the observed beneficial effects at POD3 and POD7 independent of postoperative analgesic delivery strategy.

Leave-one-out sensitivity analyses were performed to evaluate the influence of individual studies on the pooled estimates for postoperative fatigue at POD1, POD3, and POD7 (Figures S4–S6). At POD1, sequential omission of individual studies did not materially alter the pooled effect estimate, and all analyses remained statistically non-significant, with substantial heterogeneity persisting across most iterations (*I*^2^ range: 62.5–89.6%). At POD3, omission of individual studies did not change the direction or statistical significance of the pooled effect, with all analyses remaining significant and heterogeneity ranging from 23.2% to 69.5%; omission of Guo et al. (2024) [6] produced the greatest reduction in heterogeneity. Similarly, at POD7, all leave-one-out analyses continued to demonstrate significant reductions in postoperative fatigue, with heterogeneity ranging from 38.6% to 70.9%. Overall, no single study disproportionately influenced the pooled estimates, supporting the robustness and stability of the primary meta-analytic findings.

Two trials reported postoperative hospital length of stay. Sun et al. (2023) found no significant difference between groups (median 8 days [IQR 8–10] vs. 8 days [IQR 7–10]) [5]. In contrast, Lin et al. (2024) reported a shorter duration of hospitalization in the esketamine group compared with control (estimated difference −1.68 days, 95% CI −3.28 to −0.08, *p* = 0.040) [18]. Due to the limited number of studies and heterogeneity in reporting formats, meta-analysis of hospital length of stay was not performed. Several studies reported post-anesthesia care unit (PACU) or early recovery unit duration, which was similar between groups in ketamine-based regimens [15,16] and longer in one study using esketamine as part of patient-controlled intravenous analgesia [19].

The certainty of evidence was assessed using the Grading of Recommendations Assessment, Development and Evaluation (GRADE) approach (Table 2). The certainty of evidence for postoperative fatigue at POD1 was rated as low because of substantial heterogeneity (*I*^2^ = 87%) and imprecision, as the pooled confidence interval crossed the line of no effect. In contrast, the certainty of evidence for postoperative fatigue at POD3 and POD7 was rated as moderate, with downgrading primarily due to moderate to substantial statistical heterogeneity (*I*^2^ = 62% and 64%, respectively). No serious concerns regarding indirectness were identified, and sensitivity analyses supported the robustness of the pooled estimates. Publication bias was not formally assessed because fewer than 10 studies contributed to each outcome, rendering funnel plots and statistical tests for small-study effect unreliable.

## 4. Discussion

This systematic review and meta-analysis evaluated the effects of perioperative ketamine and esketamine on postoperative fatigue during the first postoperative week. The pooled analyses demonstrated no significant overall reduction in fatigue on postoperative day (POD) 1, although a significant benefit was observed in the esketamine subgroup. In contrast, ketamine and esketamine were associated with significantly lower fatigue scores at POD3 and POD7, with the greatest reduction observed at POD3. Importantly, these findings remained statistically significant following Holm–Bonferroni adjustment for multiple comparisons and were supported by sensitivity analyses excluding studies using patient-controlled intravenous analgesia (PCIA) as well as leave-one-out analyses. Collectively, these findings suggest that perioperative NMDA receptor antagonists may reduce postoperative fatigue beyond the immediate postoperative period.

The temporal pattern observed in this study is biologically plausible and consistent with the multifactorial nature of postoperative fatigue. Postoperative fatigue is a complex phenomenon influenced by surgical stress, inflammatory responses, metabolic alterations, pain, sleep disruption, and psychological factors, many of which evolve over several days following surgery rather than being confined to the immediate postoperative period [2]. Previous studies have demonstrated that postoperative fatigue frequently persists beyond the first postoperative day and may remain an important determinant of recovery during the first postoperative week [2,3]. Consequently, interventions targeting central nervous system pathways and recovery processes may be more likely to exert measurable effects several days after surgery, which could explain the more pronounced benefits observed at POD3 and POD7.

Several mechanisms may contribute to the observed reduction in postoperative fatigue. Ketamine and esketamine act primarily through antagonism of N-methyl-D-aspartate (NMDA) receptors, thereby attenuating central sensitization and reducing excitatory neurotransmission within the central nervous system [8]. Beyond their analgesic properties, these agents have been associated with improved quality of postoperative recovery, enhanced cognitive function, improved sleep quality, and reduced perioperative opioid requirements, all of which may influence fatigue perception and recovery trajectories [7,9,10,11]. In addition, ketamine and esketamine possess anti-inflammatory and antidepressant properties that may contribute to improved postoperative well-being [12]. From a mechanistic perspective, their anti-inflammatory effects may suppress perioperative neuroinflammation and downstream neuroimmune signaling, processes that have been implicated in fatigue, sickness behaviors, and persistent activation of central stress response systems [20,21]. Separately, their antidepressant actions may modulate affective and stress-regulatory neural circuits, potentially mitigating maladaptive central stress responses that contribute to the development or persistence of postoperative fatigue [22]. Through these complementary pathways, ketamine may theoretically attenuate the central stress mechanisms underlying protracted postoperative fatigue syndrome. However, it is important to acknowledge that the included studies did not directly evaluate mechanistic outcomes; therefore, these explanations remain speculative and should be interpreted cautiously.

One of the most notable findings of this meta-analysis was the greater reduction in postoperative fatigue observed among patients undergoing colorectal surgery. Significant subgroup interactions were identified at both POD3 and POD7, suggesting that the magnitude of benefit may vary according to surgical procedure. Postoperative fatigue is particularly relevant in colorectal surgery, where substantial physiological stress, inflammatory activation, and prolonged recovery are common [3]. Furthermore, fatigue has been linked to reduced physical activity and delayed mobilization after colorectal surgery, factors that may negatively affect postoperative recovery [3]. Given the central role of early mobilization and functional recovery within Enhanced Recovery After Surgery (ERAS) pathways, the observed reductions in fatigue may be especially relevant in this surgical population [23,24]. Nevertheless, these subgroup findings should be interpreted cautiously because they are based on a limited number of studies and were not derived from direct comparisons between surgical populations.

Although esketamine demonstrated a significant reduction in fatigue at POD1 whereas ketamine did not, this finding should not be interpreted as evidence of superiority. The early benefit was derived from a relatively small number of studies and was accompanied by substantial heterogeneity. Furthermore, no significant differences between ketamine and esketamine were observed at POD3 or POD7, where both agents demonstrated comparable reductions in fatigue. It is therefore possible that the apparent early effect reflects differences in study design, patient populations, or administration regimens rather than a true pharmacological advantage of esketamine. Additional adequately powered trials directly comparing ketamine and esketamine are required before firm conclusions can be drawn regarding differential efficacy.

From a clinical perspective, postoperative fatigue is increasingly recognized as an important patient-centered outcome because it may impair mobilization, delay rehabilitation, and negatively affect quality of life during recovery [3,4]. The observed reductions in fatigue scores therefore suggest a potential role for ketamine and esketamine within multimodal perioperative care strategies. However, whether improvements in fatigue scores translate into measurable improvements in objective recovery outcomes remains uncertain. The limited available evidence regarding hospital length of stay and post-anesthesia care unit recovery time was inconsistent across studies, precluding quantitative synthesis. Consequently, although reductions in fatigue are encouraging, further research is needed to establish whether these improvements lead to meaningful clinical benefits.

This study has several strengths. To our knowledge, it is the first meta-analysis specifically evaluating the effects of ketamine and esketamine on postoperative fatigue. The analysis included only randomized controlled trials, examined multiple postoperative time points, applied adjustment for multiple comparisons, and incorporated extensive sensitivity analyses. The consistency of findings across leave-one-out analyses and analyses excluding PCIA studies further strengthens confidence in the robustness of the observed effects.

Several limitations should also be considered. First, the number of available studies remained relatively small, particularly for certain subgroup analyses. Second, moderate to substantial heterogeneity was observed across several analyses, likely reflecting differences in surgical procedures, fatigue assessment tools, dosing regimens, and perioperative protocols. Third, most included studies were conducted in China, which may limit the generalizability of the findings to other healthcare settings and populations. In addition, two studies were published in Chinese and required translation for eligibility assessment and data extraction, introducing a potential risk of translation-related inaccuracies, although the impact on the pooled estimates is likely to be minimal given the predominantly quantitative nature of the extracted data. Fourth, the small number of included studies precluded formal assessment of publication bias. Finally, although statistically significant reductions in fatigue scores were observed, the clinical significance of these findings remains uncertain because no widely accepted minimal clinically important difference has been established for the Identity–Consequence Fatigue Scale or related postoperative fatigue instruments [25,26].

The certainty of evidence was rated as moderate for postoperative fatigue at POD3 and POD7, supporting a likely beneficial effect of perioperative ketamine and esketamine during the early postoperative recovery period. In contrast, the evidence for POD1 was of low certainty because of substantial heterogeneity and imprecision, suggesting that the effects in the immediate postoperative period remain uncertain. Future studies should employ standardized fatigue assessment tools, establish clinically meaningful thresholds for improvement, and determine whether reductions in postoperative fatigue translate into objective recovery outcomes such as earlier mobilization, shorter hospitalization, and improved quality of recovery. Additional high-quality randomized trials are also needed to define the optimal dose, timing, and mode of administration of ketamine and esketamine and to identify surgical populations most likely to benefit.

## 5. Conclusions

Perioperative ketamine and esketamine were associated with reduced postoperative fatigue during the first postoperative week, with the most consistent benefits observed from postoperative day 3 onward. These findings suggest a potential role for NMDA receptor antagonists within multimodal perioperative care pathways to enhance postoperative recovery. However, the clinical significance of the observed reductions in fatigue remains uncertain. Further high-quality randomized controlled trials are needed to confirm these findings, establish clinically meaningful benefit, and determine whether improvements in fatigue translate into objective recovery outcomes.

## Figures and Tables

**Table 1:** Overview of the randomized controlled trials included in the review.

Study	Country	Study Design	Procedure	Intervention Group				Control Group			
				Intervention	N	% Male	Age (Mean ± SD)	Control	N	% Male	Age (Mean ± SD)
Grady et al. (2012) * [14]	USA	RCT	Open abdominal hysterectomy	IV Ketamine 0.35 mg/kg bolus + infusion (0.2 mg/kg/h then 0.12 mg/kg/h for 24 h)	30	0%	46 ± 8	Placebo	32	0%	46 ± 8
Guo et al. (2024) ** [6]	China	RCT	Laparoscopic colorectal surgery	IV ketamine 0.3 mg/kg single bolus (5 min before incision)	44	NA	NA	Saline	44	NA	NA
Zhao et al. (2022) [15]	China	RCT	Laparoscopic colorectal surgery	IV ketamine 0.3 mg/kg single bolus (5 min before incision)	31	66%	56.4 ± 6.6	Saline	30	71%	56.4 ± 6.6
Zhao et al. (2021) [16]	China	RCT	Mastectomy	IV ketamine 0.5 mg/kg bolus + 0.25 mg/kg/h infusion until end of surgery	48	0%	50.7 ± 8.3	Saline	49	0%	50.7 ± 8.3
Verdonk et al. (2024) * [17]	France	RCT	Major orthopedic surgery	single IV ketamine 0.5 mg/kg bolus	149	37.58%	72.29 ± 7.36	Saline	143	39.16%	72.29 ± 7.36
Sun et al. (2023) [5]	China	RCT	Laparoscopic colorectal surgery	Esketamine 0.1 mg/kg IV bolus + 0.1 mg/kg/h infusion (intra-op)	32	59.38%	60.16 ± 7.97	Saline	30	56.67%	62.87 ± 8.37
Lin et al. (2024) [18]	China	RCT	Laparoscopic gastrectomy	0.5 mg/kg IV (induction) + PCIA sufentanil 2 µg/kg + esketamine 1 mg/kg (100 mL)	62	72.58%	64.30 ± 10.40	Sufentanil PCIA	62	72.58%	66.81 ± 11.27
Wang et al. (2022) ** [19]	China	RCT	Thoracoscopic lung resection	PCIA mixture contained esketamine 1.5 mg/kg + sufentanil 1.0 µg/kg	60	55%	45.6 ± 4.4	Sufentanil (PCIA)	60	48.3%	46.4 ± 4.7

Note: * Verdonk et al. (2024) [17] and Grady et al. (2012) [14] reported BFI (Brief Fatigue Inventory) and VRS (Verbal Rating Scale), respectively. Verdonk et al. (2024) [17] is a multicenter RCT. Zhao et al. (2022) [16] is a pilot RCT. RCT = randomized controlled trial. PCIA = patient-controlled intravenous analgesia. NA = not available. ** The full-text publications of Guo et al. (2024) [6] and Wang et al. (2022) [19] were published in Chinese.

**Table 2:** Certainty of evidence assessment for the effect of ketamine/esketamine on postoperative fatigue.

Outcome	No. of Studies (Participants)	Effect Estimate (SMD, 95% CI)	Certainty of Evidence	Reasons for Downgrading
**Postoperative fatigue at POD1**	6 studies (517 participants)	−0.37 (−0.87 to 0.13)	**Low** ⨁⨁◯◯	Downgraded for serious inconsistency (*I*^2^ = 87%) and imprecision (CI crossed no effect)
**Postoperative fatigue at POD3**	6 studies (552 participants)	−0.58 (−0.86 to −0.30)	**Moderate** ⨁⨁⨁◯	Downgraded for inconsistency (*I*^2^ = 62%)
**Postoperative fatigue at POD7**	6 studies (724 participants)	−0.38 (−0.65 to −0.12)	**Moderate** ⨁⨁⨁◯	Downgraded for inconsistency (*I*^2^ = 64%)

Note: Evidence was assessed using the GRADE framework across the domains of risk of bias, inconsistency, indirectness, imprecision, and publication bias. Downgrading was applied only when concerns were identified. No downgrading was performed for risk of bias or indirectness. Publication bias was not assessed because fewer than 10 studies were available for each outcome.

## Data Availability

All data analyzed in this study are derived from published studies and are available within the article and its Supplementary Materials. No new datasets were generated or analyzed.

## Supplementary Materials

The following supporting information can be downloaded at https://www.mdpi.com/article/10.3390/medicina62061156/s1, Figure S1: Sensitivity analysis excluding studies using patient-controlled intravenous analgesia (PCIA): forest plot of postoperative fatigue at postoperative day 1 (POD1). Effect sizes are presented as standardized mean differences (SMDs) with 95% confidence intervals (CIs) using a random-effects model; Figure S2: Sensitivity analysis excluding studies using patient-controlled intravenous analgesia (PCIA): forest plot of postoperative fatigue at postoperative day 3 (POD3). Effect sizes are presented as standardized mean differences (SMDs) with 95% confidence intervals (CIs) using a random-effects model; Figure S3: Sensitivity analysis excluding studies using patient-controlled intravenous analgesia (PCIA): forest plot of postoperative fatigue at postoperative day 7 (POD7). Effect sizes are presented as standardized mean differences (SMDs) with 95% confidence intervals (CIs) using a random-effects model; Figure S4: Leave-one-out sensitivity analysis for postoperative fatigue at postoperative day 1 (POD1). Sequential omission of individual studies was performed to evaluate the influence of each study on the pooled standardized mean difference (SMD) using a random-effects model; Figure S5: Leave-one-out sensitivity analysis for postoperative fatigue at postoperative day 3 (POD3). Sequential omission of individual studies was performed to evaluate the influence of each study on the pooled standardized mean difference (SMD) using a random-effects model; Figure S6: Leave-one-out sensitivity analysis for postoperative fatigue at postoperative day 7 (POD7). Sequential omission of individual studies was performed to evaluate the influence of each study on the pooled standardized mean difference (SMD) using a random-effects model; Table S1: PRISMA 2020 Checklist. Reference [27] is cited in the supplementary materials.

## Abbreviations

The following abbreviations are used in this manuscript:

ICFS	Identity–Consequence Fatigue Scale
POF	Postoperative fatigue
NMD	N-methyl-D-aspartate
RCT	Randomized controlled trial
POD	Postoperative day
MD	Mean difference
CI	Confidence interval
PACU	Post-anesthesia care unit
PCIA	Patient-controlled intravenous analgesia
ERAS	Enhanced Recovery After Surgery
RoB	Risk of bias
LOO	Leave-one-out
IQR	Interquartile range
LOS	Length of stay
CNS	Central nervous system
PROSPERO	International Prospective Register of Systematic Reviews
PRISMA	Preferred Reporting Items for Systematic Reviews and Meta-Analyses
MCID	Minimal Clinically Important Difference
GRADE	Grading of Recommendations Assessment, Development and Evaluation
