# Supplementary material for: Effect of Perioperative Ketamine and Esketamine on Postoperative Fatigue: A Systematic Review and Meta-Analysis of Randomized Controlled Trials
Source: Medicina (Kaunas). 2026 Jun 14;62(6):1156. doi: 10.3390/medicina62061156 (PMC13303731; doi:10.3390/medicina62061156)

Figure S1. Sensitivity analysis excluding studies using patient-controlled intravenous analgesia (PCIA): forest plot of postoperative fatigue at postoperative day 1 (POD1). Effect sizes are presented as standardized mean differences (SMDs) with 95% confidence intervals (CIs) using a random-effects model.

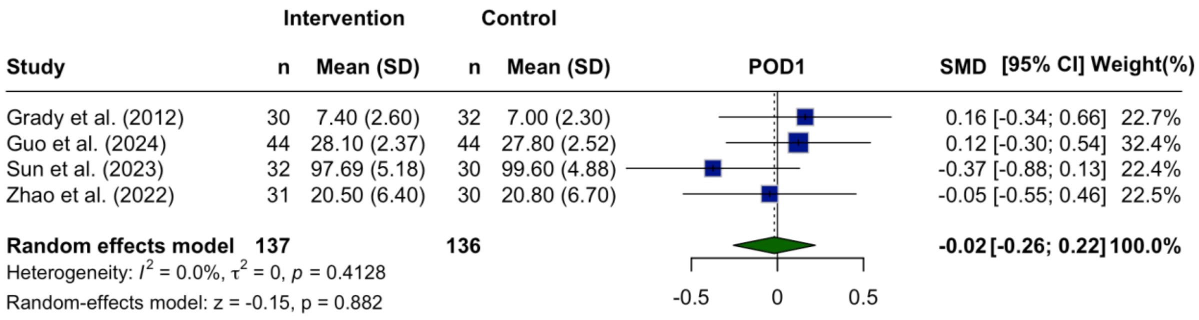

Figure S2. Sensitivity analysis excluding studies using patient-controlled intravenous analgesia (PCIA): forest plot of postoperative fatigue at postoperative day 3 (POD3). Effect sizes are presented as standardized mean differences (SMDs) with 95% confidence intervals (CIs) using a random-effects model.

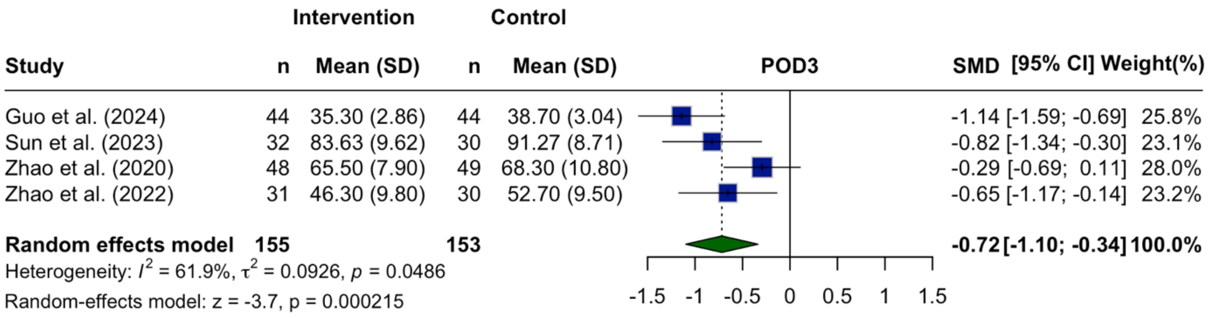

Figure S3. Sensitivity analysis excluding studies using patient-controlled intravenous analgesia (PCIA): forest plot of postoperative fatigue at postoperative day 7 (POD7). Effect sizes are presented as standardized mean differences (SMDs) with 95% confidence intervals (CIs) using a random-effects model.

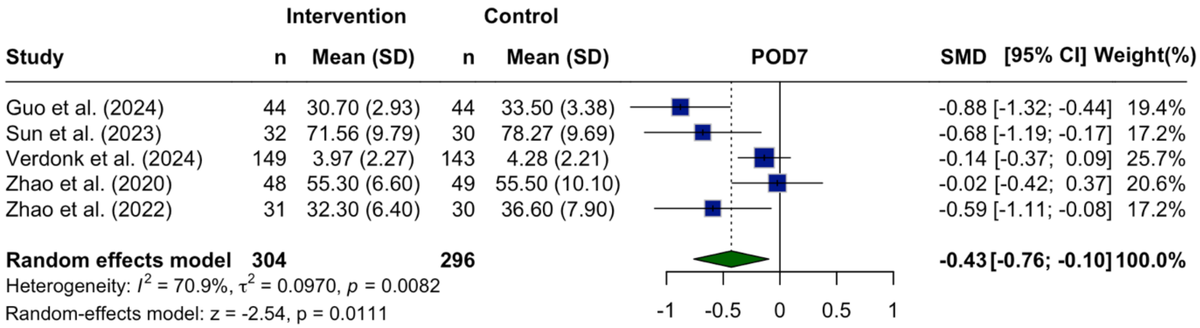

Figure S4. Leave-one-out sensitivity analysis for postoperative fatigue at postoperative day 1 (POD1). Sequential omission of individual studies was performed to evaluate the influence of each study on the pooled standardized mean difference (SMD) using a random-effects model.

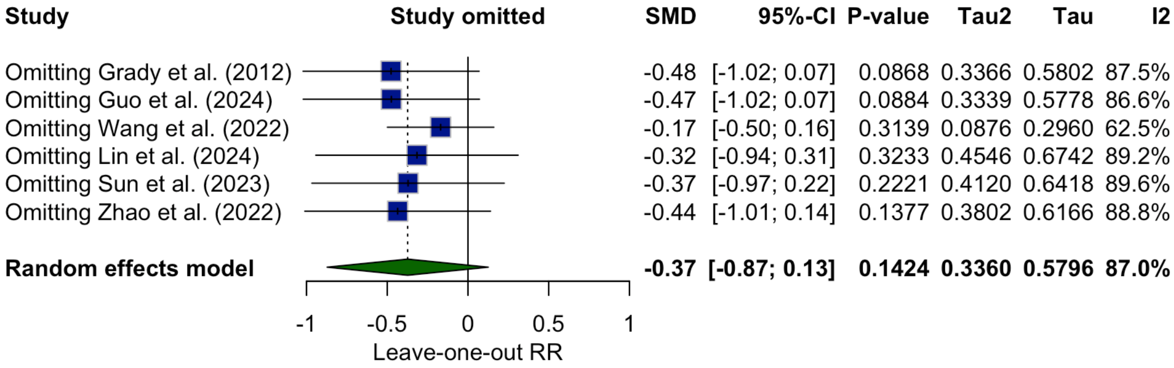

Figure S5. Leave-one-out sensitivity analysis for postoperative fatigue at postoperative day 3 (POD3). Sequential omission of individual studies was performed to evaluate the influence of each study on the pooled standardized mean difference (SMD) using a random-effects model.

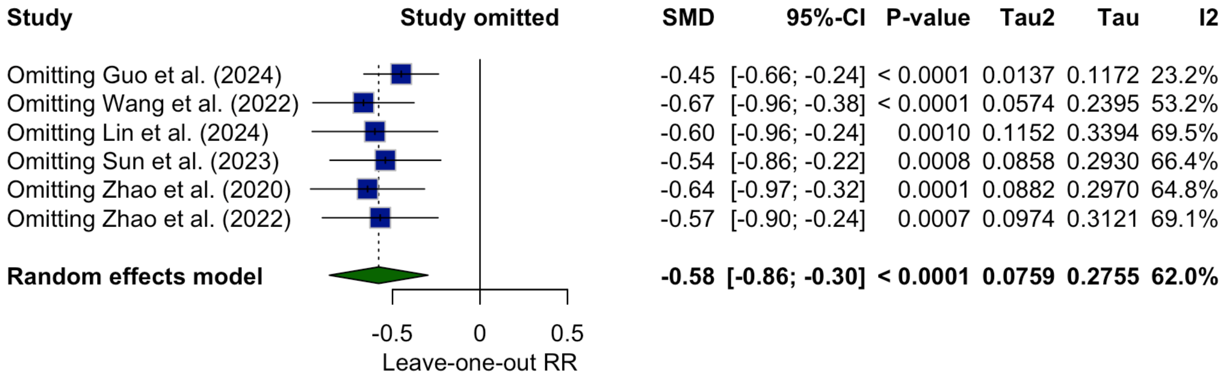

Figure S6. Leave-one-out sensitivity analysis for postoperative fatigue at postoperative day 7 (POD7). Sequential omission of individual studies was performed to evaluate the influence of each study on the pooled standardized mean difference (SMD) using a random-effects model.

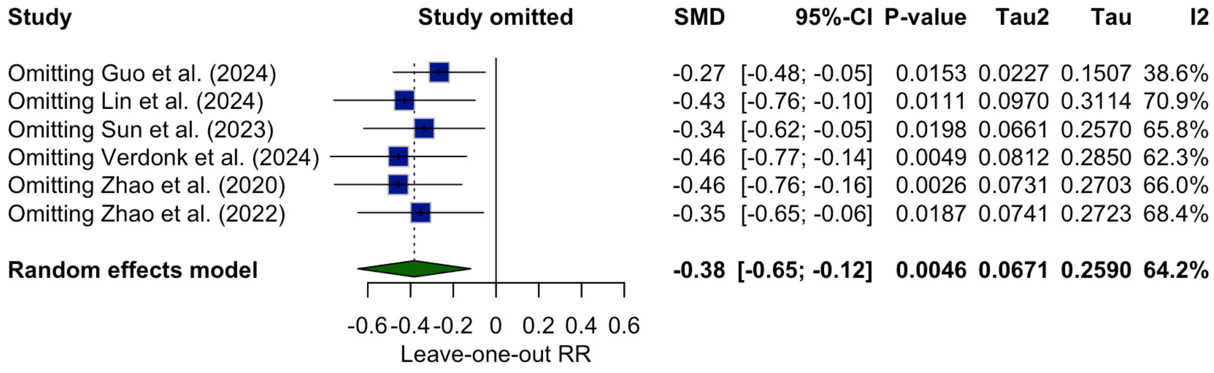

Supplement: Supplementary file 1 [file medicina-62-01156-s001.zip › medicina-4324308-figures.pdf]
